# Abusive Head Trauma in Infants During the COVID-19 Pandemic in the Paris Metropolitan Area

**DOI:** 10.1001/jamanetworkopen.2022.26182

**Published:** 2022-08-30

**Authors:** Alina-Marilena Lãzãrescu, Sandro Benichi, Thomas Blauwblomme, Kévin Beccaria, Marie Bourgeois, Charles-Joris Roux, Estelle Vergnaud, Juliette Montmayeur, Philippe Meyer, Jérémie F. Cohen, Martin Chalumeau, Flora Blangis, Gilles Orliaguet

**Affiliations:** 1 Department of Pediatric Anesthesia and Intensive Care, Assistance Publique–Hôpitaux de Paris, Necker-Enfants Malades Hospital, Université Paris Cité, Paris, France; 2 Department of Pediatric Neurosurgery, Assistance Publique–Hôpitaux de Paris, Necker-Enfants Malades Hospital, Université Paris Cité, Paris, France; 3 Department of Pediatric Radiology, Assistance Publique–Hôpitaux de Paris, Necker-Enfants Malades Hospital, Université Paris Cité, Paris, France; 4 Obstetrical, Perinatal and Pediatric Epidemiology Research Team, Centre of Research in Epidemiology and Statistics, Université Paris Cité, Institut National de la Santé et de la Recherche Medicale, Paris, France; 5 Child Protection Unit, Department of General Pediatrics and Pediatric Infectious Diseases, Assistance Publique–Hôpitaux de Paris, Necker-Enfants Malades Hospital, Université Paris Cité, Paris, France; 6 Unit EA7323 Evaluation of Therapeutics and Pharmacology in Perinatality and Pediatrics, Université Paris Cité, Paris, France

## Abstract

**Importance:**

The COVID-19 pandemic and the containment and mitigation measures taken were feared to be associated with increased child abuse.

**Objective:**

To investigate the trend of abusive head trauma (AHT) incidence and severity in infants during the COVID-19 pandemic.

**Design, Setting, and Participants:**

In a time-series analysis of a longitudinal, population-based, cohort study, all consecutive cases of AHT in infants younger than 12 months old referred between January 2017 and December 2021 to Necker Hospital for Sick Children, the single regional pediatric neurosurgery center for the Paris metropolitan area, were included. AHT was defined as 1 or more subdural hemorrhage and a positive multidisciplinary evaluation after a social, clinical, biological, and radiological workup. Data were analyzed from January to March 2022.

**Main Outcomes and Measures:**

The primary outcome was the monthly incidence of AHT, which was analyzed using Poisson regression modeling. Secondary outcomes included mortality and severe morbidity and were studied with logistic and linear regressions. The monthly incidence of neurosurgical interventions for hydrocephalus was used as a control series.

**Results:**

Among the 99 included infants with AHT (median [IQR] age, 4 [3-6] months; 64 boys [65%]), 86 of 99 (87%) had bridging vein thrombosis, 74 of 99 (75%) had retinal hemorrhages, 23 of 72 (32%) had fractures, 26 of 99 (26%) had status epilepticus, 20 of 99 (20%) had skin injuries, 53 of 99 (54%) underwent neurosurgical interventions, and 13 of 99 (13%) died. Compared with the prepandemic period (2017-2019), AHT incidence was stable in 2020 (adjusted incidence rate ratio, 1.02; 95% CI, 0.59-1.77) and then significantly increased in 2021 (adjusted incidence rate ratio, 1.92; 95% CI, 1.23-2.99). The severity of AHT worsened in 2021 in terms of mortality (odds ratio 9.39; 95% CI, 1.88-47.00). Other secondary outcomes and the control series were not significantly modified.

**Conclusions and Relevance:**

In this cohort study, a marked increase in AHT incidence and severity occurred during the COVID-19 pandemic in the Paris metropolitan area. These results suggest the need for clinical awareness and preventive actions.

## Key Points

**Question:**

Did the incidence of abusive head trauma in infants in the Paris metropolitan area increase during the first 2 years of the COVID-19 pandemic (2020-2021) compared with the prepandemic period (2017-2019)?

**Findings:**

In a time-series analysis of a cohort study including 99 infants, abusive head trauma incidence was stable in 2020 and then nearly doubled, a significant increase, in 2021.

**Meaning:**

These findings suggest that the marked increase in abusive head trauma incidence during the COVID-19 pandemic in the Paris metropolitan area should prompt clinical awareness and preventive actions.

## Teaser

This cohort study evaluates whether the incidence of abusive head trauma in infants in the Paris metropolitan area increased during the first 2 years of the COVID-19 pandemic (2020-2021) compared with the prepandemic period (2017-2019).

## Introduction

Abusive head trauma (AHT), the most severe form of child abuse and neglect (CAN), is defined as cerebral, cranial, and/or spinal injuries that result from inflicted injury to infants and young children.^1,2,3^ AHT’s main features are subdural hemorrhage (SDH) and bridging vein thrombosis, which are usually associated with retinal hemorrhage and, less frequently, fractures.^4,5^ AHT is the most frequent cause of traumatic death in infants in high-income countries,^1,2^ and nonlethal forms are associated with severe long-term morbidity, such as neurodevelopmental impairment (microcephaly, epilepsy, motor and visual deficiencies, language disorders, intellectual disability, and behavioral abnormalities) leading to severe lifelong disabilities.^6^ Among the known risk factors of AHT, several could have seen their prevalence modified by the COVID-19 pandemic and the containment and mitigation measures taken to reduce the spread of the disease^7,8^: psychosocial distress (economic loss and unemployment, frustration intolerance, adult psychiatric disorders, and intimate partner violence),^9,10^ lifestyle changes (remote work from home in small collective housing, school and childcare facility closures, complete national lockdowns, and curfews),^11^ and disorganized social services.^9,12^ Concerns were raised early regarding a potentially marked increase in CAN (notably AHT) in association with the COVID-19 pandemic and related containment and mitigation measures.^13,14,15^

Studies evaluating the association of the COVID-19 pandemic and containment and mitigation measures with the incidence and severity of CAN and AHT led to conflicting conclusions, varying from a significant increase,^16,17,18,19^ a significant decrease,^20,21,22^ or no change^23,24,25,26^ (eTable 1 in the Supplement). These contradictory findings may be secondary to regional variability in the prepandemic epidemiology of CAN and social programs and the COVID-19 pandemic intensity and containment and mitigation measures taken. Inconsistencies may also be due to study design considerations, such as variability in the definitions of CAN and AHT, case identification strategy, and statistical approaches used. To our knowledge, no robust population-based, time-series analysis of AHT incidence in a region with a high incidence of COVID-19 and major containment and mitigation measures has been reported.

The Paris metropolitan area is of particular interest to study the association between the COVID-19 pandemic and AHT incidence. Indeed, it is a densely populated region where two-thirds of the population lives in small collective housing, and it faced strong COVID-19 pandemic waves that led to prolonged lockdowns and curfews, remote work obligations, and daycare center closures.^27,28^ Furthermore, the longtime health care organization for AHT in this region helps conduct population-based studies. The present study aimed to investigate the trend of AHT incidence and severity in infants in the Paris metropolitan area during the 2 first years of the COVID-19 pandemic compared with the prepandemic period.

## Methods

### Study Design

We followed the Strengthening the Reporting of Observational Studies in Epidemiology (STROBE) reporting guideline to report this study.^29^ We conducted a time-series analysis of a longitudinal, population-based, cohort study using patient-level electronic health records (EHRs) in a tertiary university hospital in Paris, France. Necker Hospital for Sick Children is the single regional pediatric neurosurgery referral facility for the Paris metropolitan area (12.2 million inhabitants, including 158 000 infants younger than 1 year). The local ethics committee approved the study and waived the need for parental written informed consent because all parents were informed of their tacit nonopposition to the use of routinely collected data.

### Participants and Diagnostic Workup

We included all consecutive cases of AHT with SDH in infants younger than 12 months old referred between January 1, 2017, and December 31, 2021.^30^ AHT with SDH was chosen because SDH is the most common intracranial lesion in young infants with AHT.^31^ This age limit was chosen because, although the incidence of CAN is similar immediately before and after this cutoff, AHT with SDH mainly occurs before this age limit.^32^ Two independent physicians (A.-M.L. and S.B.) cross-examined the hospital EHR database in which all medical records are being stored, using the term SDH as a search word, as well as the local prospective registry of reports to judicial authorities to identify potentially eligible cases. The hospital EHR database and the registry of reports to judicial authorities have been used in routine medical care since 2015^30^ and 2010, respectively. They also extracted the monthly number of neurosurgical interventions for hydrocephalus (endoscopic third ventriculostomy or ventriculoperitoneal shunt) nonrelated to AHT or postneonatal meningitis in infants younger than 12 months over the same period. Hydrocephalus was selected as the control series because of similarities with AHT in terms of organization of care in the Paris metropolitan area and age range (with a median age at diagnosis of 2 months), and because its incidence is not likely to have been influenced by the pandemic and the containment and mitigation measures; the main causes of hydrocephalus in this age group are intraventricular hemorrhage in extremely premature neonates, arachnoid cyst, and spinal dysraphism.^33^

For the present study, AHT was defined as 1 or more SDH and a positive multidisciplinary evaluation after a complete social, clinical, biological, and radiological workup, as suggested in the literature^34^ and in the national clinical guidelines.^35^ According to the national guidelines,^35^ these criteria correspond to the highest degree of suspicion of AHT. During the study period, the multidisciplinary team involved in the evaluation used these criteria consistently and the routine local protocol called for all infants to have a review of their medical history, examination for signs of inflicted skin injuries (bruises and hematoma in a noncruising child, burns, abrasions, lacerations, and scars), head and cervical magnetic resonance imaging (MRI) to assess potential brain and medullar damages, electroencephalogram, eye fundus examination performed by an experienced ophthalmologist within the first 48 hours after admission to look for retinal hemorrhages, radiological skeletal surveys to look for fractures, and blood tests to look for a hemostasis disorder. Some infants did not undergo all of these procedures because they died early or were clinically unstable (eg, refractory status epilepticus).

For included cases, the following data were independently extracted from the EHR by 2 experienced physicians (A.-M. L. and S.B.): parental residential zip code, age at diagnosis, sex, inflicted lesions (ie, bridging vein thrombosis, retinal hemorrhages, fractures, and skin injuries) and short-term consequences of AHT (Glasgow Coma Scale score at arrival in the neurosurgery department, status epilepticus, refractory status epilepticus, ischemic lesions on MRI, neurosurgical interventions, admission to the pediatric intensive care unit, and death before discharge). We excluded infants whose parents’ residence was not in the Paris metropolitan area (11 participants).

### COVID-19 Containment and Mitigation Measures

In France, several measures were implemented in 2020 and 2021 to contain and mitigate the COVID-19 pandemic. A first lockdown occurred from March 17 to May 11, 2020, a second from October 30 to December 15, 2020, and a third from April 3 to May 3, 2021. Other measures included curfews and the closure of daycare centers and schools during the first lockdown. Daycare centers, childminders, and schools only continued to look after the children of the parents working in essential services (eg, health care services). Schools reopened progressively from May 11, 2020, until the summer break. Remote work from home was mandatory during the first lockdown, except for essential services, and strongly recommended during the second and third lockdowns. No national prevention campaign against AHT was implemented in France until January 2022.

### Statistical Analysis

Our primary outcome was the monthly incidence of AHT, which was analyzed using Poisson regression modeling, accounting for seasonality, after checking for the absence of overdispersion. Seasonality was considered by including harmonic terms (sines and cosines) with 12-month periods.^36^ We pooled the years 2017 to 2019 (prepandemic period) to increase the statistical power and distinguished the years 2020 and 2021, where several lockdowns, containment, and mitigation measures were implemented, as noted already. The monthly number of neurosurgical interventions for hydrocephalus (nonrelated to AHT or postneonatal meningitis) was analyzed using the same modeling strategy. Secondary outcomes included markers of the severity of the inflicted lesions (ie, bridging vein thrombosis, retinal hemorrhages, fractures, and skin injuries) and short-term consequences of AHT.^37,38,39^ These outcomes were analyzed with logistic and linear regression modeling. Two-sided likelihood ratio tests were used for Poisson and logistic regression modeling, and 2-sided F-tests for linear regression modeling, with *P* < .05 considered statistically significant.

We conducted 2 sensitivity analyses. In the first, we looked for a time progression in the incidence of AHT by analyzing the pandemic periods as 4 segments of 6 months each instead of 1-year segments. In the second, we also included in the analyses infants whose parents’ residence was not in the Paris metropolitan area (11 participants). We used R statistical software version 4.1.1 (R Project for Statistical Computing) and Stata/SE statistical software version 15.1 (StataCorp) for all analyses. Data were analyzed from January to March 2022.

## Results

### Participants

Among the 99 infants with a confirmed diagnosis of AHT included in the study, the median (IQR) age was 4 (3-6) months, 64 were boys (65%). Among the included infants, 87% (86 of 99 participants) had bridging vein thrombosis (77% multiple thrombosis; 66 of 86 participants), 75% (74 of 99 participants) had retinal hemorrhages (82% bilateral; 61 of 74 participants), 32% (23 of 72 participants) had fractures, and 20% (20 of 99 participants) had skin injuries. The median (IQR) Glasgow Coma Scale score at arrival in the neurosurgery department was 14 (10-15), 26% (26 of 99 participants) of the patients had status epilepticus, 24% (24 of 99 participants) had refractory status epilepticus, 30% (29 of 97 participants) had ischemic lesions on MRI, 54% (53 of 99 participants) underwent neurosurgical interventions (79% [42 of 53 participants] subduroperitoneal shunt and 21% [11 of 53 participants] external subdural drainage), 29% (29 of 99 participants) were admitted to the pediatric intensive care unit, and 13% (13 of 99 participants) died before discharge. All the cases included in this analysis were reported to judicial authorities. During the study period, 231 infants younger than 12 months had neurosurgical interventions for hydrocephalus not related to AHT or postneonatal meningitis (control series).

### Association of COVID-19 Pandemic With the Incidence and Severity of AHT

The mean (SD) monthly incidence of AHT varied by years: 1.1 (1.2) cases in 2017, 1.5 (2.3) cases in 2018, 1.6 (1.4) cases in 2019, 1.4 (1.4) cases in 2020, and 2.7 (2.1) cases in 2021. Compared with the prepandemic period (2017-2019), AHT incidence was stable in 2020 (adjusted incidence rate ratio [aIRR], 1.02; 95% CI, 0.59-1.77) and then nearly doubled in 2021 (aIRR, 1.92; 95% CI, 1.23-2.99; *P* = .02) (Figure). There was no significant difference in the incidence of neurosurgical interventions for hydrocephalus in 2020 and 2021 compared with the prepandemic period (Figure). The severity of AHT significantly worsened in 2021 in terms of mortality (odds ratio, 9.39; 95% CI, 1.88-47.00; *P* = .007); other secondary outcomes were not significantly modified during the pandemic (Table).

**Figure.**
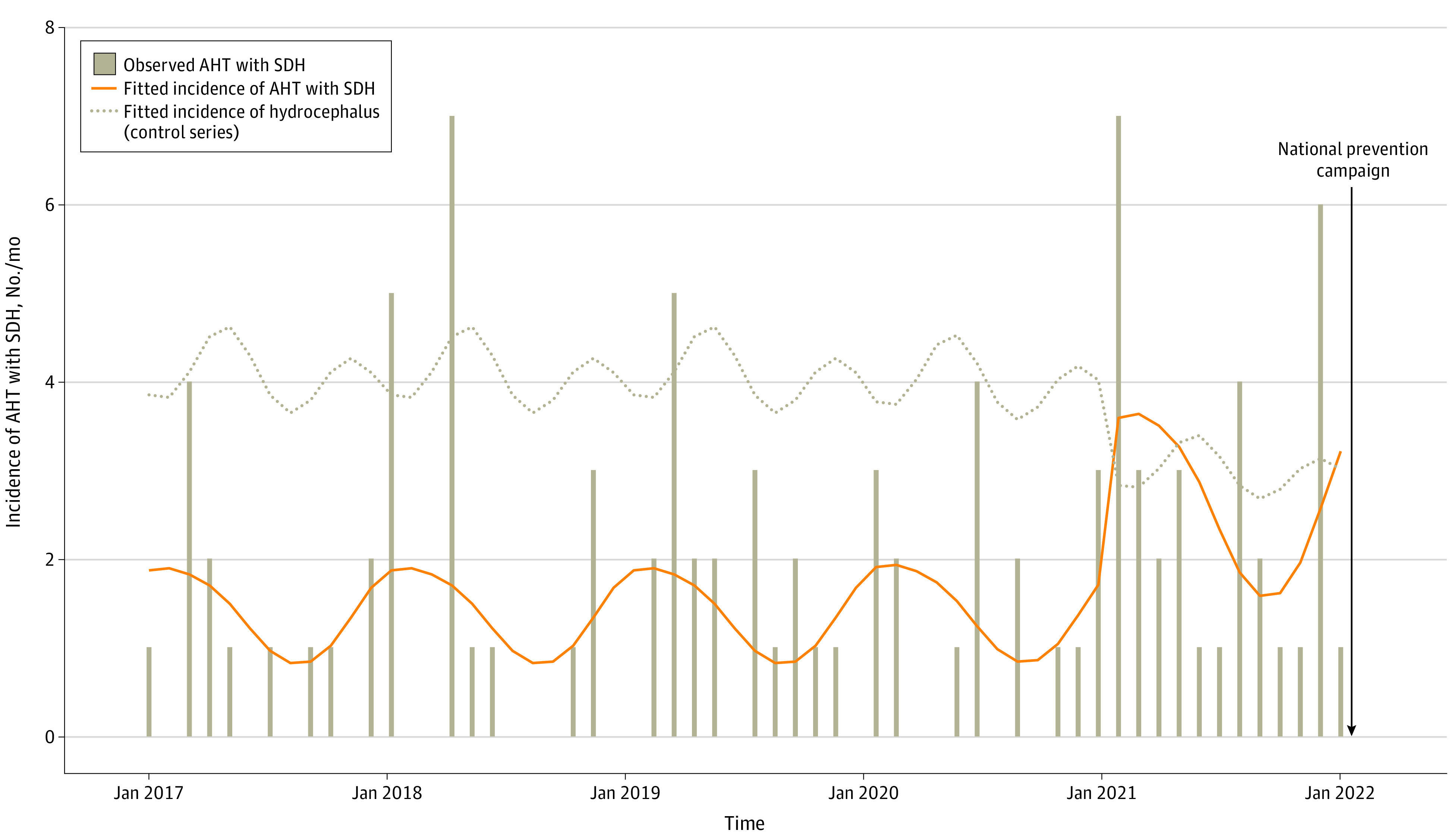
Abusive Head Trauma (AHT) Incidence (Number of Cases Per Month) in Infants in the Paris Metropolitan Area The bars show the observed data. The orange line shows the fitted values of abusive head trauma, and the dotted line shows the fitted values of hydrocephalus (Poisson regression modeling adjusted on seasonality). SDH indicates subdural hemorrhage.

**Table.** Comparison of the Frequencies of the Children’s Inflicted Lesions and Outcomes in 2020 and 2021 vs the Prepandemic Period (2017-2019)

Outcome and years	Patients, No./total No. (%)	OR (95% CI)	*P* value^a^
Bridging vein thrombosis			
2017-2019	43/50 (86)	1 [Reference]	.96
2020	15/17 (88)	1.22 (0.23 to 6.54)	
2021	28/32 (88)	1.14 (0.31 to 4.25)	
Retinal hemorrhages			
2017-2019	35/50 (70)	1 [Reference]	.29
2020	15/17 (88)	3.21 (0.65 to 15.83)	
2021	24/32 (75)	1.29 (0.47 to 3.51)	
Fractures			
2017-2019	15/45 (33)	1 [Reference]	.89
2020	2/8 (25)	0.67 (0.12 to 3.71)	
2021	6/19 (32)	0.92 (0.29 to 2.91)	
Skin injuries			
2017-2019	7/50 (14)	1 [Reference]	.28
2020	4/17 (24)	1.89 (0.48 to 7.48)	
2021	9/32 (28)	2.40 (0.79 to 7.29)	
Glasgow Coma Scale score^b^			
2017-2019 (n = 50)	14 (11 to 15)	Reference	.90
2020 (n = 32)	14 (11 to 15)	−0.18 (−2.16 to 1.79)	
2021 (n = 32)	14 (9 to 15)	−0.36 (−1.95 to 1.23)	
Status epilepticus			
2017-2019	10/50 (20)	1 [Reference]	.21
2020	4/17 (24)	1.23 (0.33 to 4.60)	
2021	12/32 (38)	2.40 (0.89 to 6.50)	
Refractory status epilepticus			
2017-2019	9/50 (18)	1 [Reference]	.25
2020	4/17 (24)	1.40 (0.37 to 5.31)	
2021	11/32 (34)	2.39 (0.86 to 6.66)	
Ischemic lesions on magnetic resonance imaging			
2017-2019	11/48 (23)	1 [Reference]	.24
2020	5/17 (29)	1.40 (0.40 to 4.85)	
2021	13/32 (41)	2.30 (0.87 to 6.10)	
Neurosurgical interventions			
2017-2019	31/50 (62)	1 [Reference]	.17
2020	9/17 (53)	0.69 (0.23 to 2.09)	
2021	13/32 (41)	0.42 (0.17 to 1.04)	
Pediatric intensive care unit			
2017-2019	13/50 (26)	1 [Reference]	.72
2020	5/17 (29)	1.19 (0.35 to 4.02)	
2021	11/32 (34)	1.49 (0.57 to 3.91)	
Death			
2017-2019	2/50 (4)	1 [Reference]	.007
2020	2/17 (12)	3.20 (0.41 to 24.70)	
2021	9/32 (28)	9.39 (1.88 to 47.00)	

^a^ *P* values were calculated using univariable logistic or linear regression.

^b^ Data are median (IQR) and β (95% CI).

### Sensitivity Analyses

When using semesters instead of 1-year periods in 2020 to 2021, the incidence of AHT increased 2.5 times from July to December 2021 (aIRR, 2.46; 95% CI, 1.28-4.72) compared with the prepandemic period (eTable 2 in the Supplement). When including the 11 infants whose parents’ residence was not in the Paris metropolitan area, the Poisson model yielded similar results (eTable 2 in the Supplement).

## Discussion

### Main Findings

The containment and mitigation measures implemented during the COVID-19 pandemic have saved approximately 690 000 adult lives in France up to May 2020, notably among the elderly.^40^ The COVID-19 pandemic and the containment and mitigation measures taken were also associated with severe adverse social, physical, and mental health effects among the pediatric population, including food insecurity, prolonged screen time, reduced physical activity, obesity,^41^ depression and anxiety,^42^ suicide ideation and attempts,^43^ and anorexia,^44^ and were feared to increase the risk of CAN.^14,15^ Indeed, the COVID-19 pandemic and the containment and mitigation measures taken deteriorated the psychosocial situation of adults, increased the periods where parents or guardians were at home for a prolonged time with their children, and reduced the intensity of prevention and early detection programs of CAN. In this first population-based cohort study, we found that the COVID-19 pandemic and the containment and mitigation measures were also associated with a marked increase of the incidence (aIRR 1.92; 95% CI, 1.23-2.99) and severity (mortality odds ratio, 9.39; 95% CI, 1.88-47.00) of AHT in the Paris metropolitan area.

### Interpretation and Comparison With Previous Findings

Parental distress associated with social isolation,^45^ economic loss and unemployment, mental disorders such as acute and posttraumatic stress disorders, and depression or suicidal behaviors^46,47,48^ are known risk factors for CAN and have been accentuated as a result of the COVID-19 pandemic and the measures implemented to reduce the spread of the disease.^46,47,48,49^ The design of our study did not allow deciphering the respective roles of the pandemic and these measures. However, given that this epidemic of AHT did not happen during the first year of the pandemic where the containment and mitigation measures were maximum, but during its second year, and notably the fourth semester of 2021, we can hypothesize that the causal pathway toward increased AHT incidence is more secondary to the accumulation of psychosocial distress over time than to the lockdowns. Hypotheses regarding the role of a reduction of CAN prevention and early detection programs are more hazardous given their potential long-lasting effects.

Our findings are consistent with previous studies reporting an increase in CAN during the COVID-19 pandemic. For example, Loiseau et al^19^ and Kovler et al^17^ reported an increase in physical abuse of 50% and 130%, respectively. Also, Sidpra et al^16^ reported an increase of 1500% in the incidence of AHT in children. Other studies reported no change in the incidence of CAN^23,24,25,26^ or a decrease up to 50% in emergency department visits related to CAN^21^ and 50% decrease of children with AHT^20^ (eTable 1 in the Supplement). This discrepancy can be secondary to the specific situation of the Paris metropolitan area (a region heavily affected by the COVID-19 pandemic, with compulsory mitigation measures, and with the majority of the population living in small collective housing). It would be interesting to investigate whether the increase was geographically heterogenous within this area and if it was associated with specific living conditions. The discrepancy can also be secondary to the definitions and design of these previous studies. Indeed, these studies^20,22,23,24,26^ compared the incidence of CAN or AHT during the second and/or third quarter of 2020 with the prepandemic period but did not explore the incidence of AHT in 2021, making it impossible to assess the medium-term consequences of the pandemic and the mitigation measures taken. The increase in mortality could be explained by an increased delay between the trauma and the first medical examination,^50^ leading to untreated seizures and additional brain damage.^51^ Moreover, none of these previous studies was population-based, thus failing to consider every case of CAN. Furthermore, half of them used administrative data,^20,21,22^ which means that the incidence of CAN was conditional on the coding errors of discharge diagnoses. Finally, none of them used time-series analyses, thus not allowing assessment of the time trend of CAN, but only a comparison of its average incidence over 2 periods.

### Strengths and Limitations

Our study has several strengths. First, we performed a regional, population-based study as all children suspected of AHT in the Paris metropolitan area must be referred to Necker Hospital for Sick Children. This was confirmed through the stability of the incidence of neurosurgical interventions for hydrocephalus during the study period, to which the same organization of care applies. Moreover, the definition of AHT (≥1 SDH and a positive multidisciplinary evaluation) and the strategy to identify eligible cases (double-check between the search in the hospital EHR database and the registry of reports to judicial authorities) were both highly sensitive and specific. Then, we analyzed AHT incidence over an extensive period, not only over the few months of lockdown. This allowed us to study the medium-term consequences of the pandemic and all the containment and mitigation measures implemented in France over the last 2 years that have had potential repercussions on families and on the increase in CAN, not only the lockdown periods. Sensitivity analyses by 6-month increments in 2020 and 2021 reported the same results, thus confirming the main analyses.

Our study has several limitations. First, although we included all severe AHT cases hospitalized in the Paris metropolitan area, mild cases of AHT might have been undiagnosed or not referred to our reference center. Other children with AHT might have been misclassified as having accidental trauma or might have died before diagnosis and been classified as cases of sudden infant death, although the number of infanticide cases over the study period remained stable according to the national registry.^52^ Furthermore, we specifically included infants with AHT with SDH, and our findings may not apply to the very few infants with AHT without SDH. This selection bias might have underestimated the actual incidence of AHT, confirming the increased incidence of AHT. Second, we were unable to study the long-term consequences (eg, disabilities) associated with AHT because of the time limitation of the study. As the number of deaths from AHT dramatically increased in 2021 compared with previous years, we believe that the long-term consequences might have also increased in number and severity. Third, the number of births in France during the COVID-19 pandemic period may be associated with the incidence of AHT. However, the decrease in crude birth rate during this period^53^ confirms the increase in the incidence of AHT in infants. Fourth, we focused only on the incidence of AHT. It would have been interesting to study other types of CAN (eg, nonaccidental fractures) in the same population over 2020 and 2021; these have also likely increased. Additionally, we could only make hypotheses regarding the causes of the increase in AHT incidence.^49^

## Conclusions

We found a marked increase in incidence and severity of AHT with SDH during the COVID-19 pandemic period in the Paris metropolitan area compared with the prepandemic period. Although the containment and mitigation measures were necessary to reduce the spread of COVID-19, they may have had unintended health consequences for children, such as an increase in AHT. These results suggest the need for clinical awareness and preventive actions.

## Notes

Supplement. **eTable 1. **Review of Studies on Child Abuse During the COVID-19 Pandemic Period **eTable 2. **Sensitivity Analysis
eReferences
