# Supplementary material for: Abusive Head Trauma in Infants During the COVID-19 Pandemic in the Paris Metropolitan Area
Source: JAMA Netw Open. 2022 Aug 30;5(8):e2226182. doi: 10.1001/jamanetworkopen.2022.26182 (PMC9428740; doi:10.1001/jamanetworkopen.2022.26182)
Supplement: Supplement. — eTable 1. Review of Studies on Child Abuse During the COVID-19 Pandemic Period eTable 2. Sensitivity Analysis eReferences [file jamanetwopen-e2226182-s001.pdf]

Supplemental Online Content

Lãzãrescu A-M, Benichi S, Blauwblomme T, et al. Abusive head trauma in infants during the COVID-19 pandemic in the Paris metropolitan area. *JAMA Netw Open*. 5(8):e2226182. doi:10.1001/jamanetworkopen.2022.26182

eTable 1. Review of Studies on Child Abuse During the COVID-19 Pandemic Period

eTable 2. Sensitivity Analysis

eReferences

This supplemental material has been provided by the authors to give readers additional information about their work.

© 2022 Lãzãrescu A-M et al. *JAMA Network Open.*

eTable 1. Review of Studies on Child Abuse During the COVID-19 Pandemic Period

Authors	Country	Recruitment	Number of centers	Period analyzed	Reference period	Type of abuse	Number of children (2020)	Risk
**Davidson**[1]	UK	Local	1	Quarter 2 2020	2019	NAI	4	Equal
**Kaiser**[2]	USA	National	52	Quarters 1 to 3 2020	2017-2019	NAI	621	-25%
**Kovler**[3]	USA	Local	1	Quarter 2 2020	2018-2019	NAI	8	+128%
**Loiseau**[4]	France	National	-	Quarter 2 2020	2017-2019	NAI	476	+40%
**Maassel**[5]	USA	National	49	Quarters 2 and 3 2020	2017-2019	AHT	127	-50%
**Massiot**[6]	France	Local	1	Quarters 2 and 3 2020	2018-2019	NAI	358	Equal
**Sanford**[7]	USA	Local	2	Quarter 2 2020	2015-2019	NAI	392	Equal
**Sidpra**[8]	UK	Local	1	Quarter 2 2020	2017-2019	AHT	10	+1500%
**Swedo**[9]	USA	National	2970	Quarter 2 2020	2019	NAI	Not available	Absolute decrease / increase in % of total

NAI: non-accidental injury; AHT: abusive head trauma

© 2022 Lãzãrescu A-M et al. *JAMA Network Open.*

eTable 2. Sensitivity Analysis

	**Main analysis**		**Sensitivity analysis**				
	**Analysis by year, with infants living in Ile-de-France**		**Analysis by year, with infants living in and outside Ile-de-France**		**Analysis by semester**		
	**aIRR***	**p**	**aIRR***	**p**		**aIRR***	**p**
**Periods**		0.02		0.007	**Periods**		0.054
**2017-2019**	Ref		Ref		**2017-2019**	Ref	
**Jan-Dec 2020**	1.02 [0.59 - 1.77]		0.96 [0.57 - 1.64]		**Jan-Jun 2020**	0.95 [0.47 - 1.91]	
					**July-Dec 2020**	1.15 [0.49 - 2.68]	
**Jan-Dec 2021**	1.92 [1.23 - 2.99]		1.93 [1.27 - 2.93]		**Jan-Jun 2021**	1.61 [0.90 - 2.87]	
					**July-Dec 2021**	2.46 [1.28 - 4.72]	

* Poisson regression modeling adjusted on seasonality
aIRR: adjusted incidence rate ratio

© 2022 Lãzãrescu A-M et al. *JAMA Network Open.*

eReferences

1. Davidson M, Saran S, Mansoor Q, *et al.* Non-accidental injury and abusive head trauma in children–is the COVID-19 pandemic impacting teesside differently? *BMJ Paediatr Open* 2021; **5**: A130-A.
2. Kaiser SV, Kornblith AE, Richardson T, *et al.* Emergency visits and hospitalizations for child abuse during the COVID-19 pandemic. *Pediatrics* 2021; **147**: e2020038489.
3. Kovler ML, Ziegfeld S, Ryan LM, *et al.* Increased proportion of physical child abuse injuries at a level I pediatric trauma center during the Covid-19 pandemic. *Child Abuse Negl* 2021; **116**: 104756.
4. Loiseau M, Cottenet J, Bechraoui-Quantin S, *et al.* Physical abuse of young children during the COVID-19 pandemic: Alarming increase in the relative frequency of hospitalizations during the lockdown period. *Child Abuse Negl* 2021; **122**: 105299.
5. Maassel NL, Asnes AG, Leventhal JM, *et al.* Hospital admissions for abusive head trauma at children's hospitals during COVID-19. *Pediatrics* 2021; **148**: e2021050361.
6. Massiot L, Launay E, Fleury J, *et al.* Impact of COVID-19 pandemic on child abuse and neglect: A cross-sectional study in a French Child Advocacy Center. *Child Abuse Negl* 2021: 105443.
7. Sanford EL, Zagory J, Blackwell J-M, *et al.* Changes in pediatric trauma during COVID-19 stay-at-home epoch at a tertiary pediatric hospital. *J Pediatr Surg* 2021; **56**: 918-22.
8. Sidpra J, Abomeli D, Hameed B, *et al.* Rise in the incidence of abusive head trauma during the COVID-19 pandemic. *Arch Dis Child* 2021; **106**: e14.
9. Swedo E, Idaikkadar N, Leemis R, *et al.* Trends in US emergency department visits related to suspected or confirmed child abuse and neglect among children and adolescents aged< 18 years before and during the COVID-19 pandemic—United States, January 2019–September 2020. *MMWR Morb Mortal Wkly Rep* 2020; **69**: 1841.

© 2022 Lãzãrescu A-M et al. *JAMA Network Open.*
